# Assessment of the remineralizing potential of nano-bioactive glass, nano-hydroxyapatite, and sodium fluoride on artificial carious lesions in primary teeth

**DOI:** 10.1186/s12903-026-08279-6

**Published:** 2026-04-21

**Authors:** Hanin Ahmed ElZohairy, Basma Mahmoud Nagi, Gehan Gaber Allam

**Affiliations:** Department of Pediatric Dentistry and Dental Public Health, Faculty of Dentistry, Ain Shams University, African Unity St., Cairo, 11566 Egypt

**Keywords:** Sodium Fluoride, Varnish, n-BAG, n-HAP, Enamel remineralization, Primary teeth, SEM-EDX, PLM

## Abstract

**Background:**

Tooth decay represents a dynamic balance characterized by alternating cycles of demineralization and remineralization, though loss of minerals can be reversed in its early stages. Recently, nanomaterials have been widely explored in dentistry due to their remarkable characteristics and enhanced remineralization potential. This study aimed to assess the remineralization performance of three varnishes on artificial enamel lesions in primary teeth: commercial fluoride varnish (5% NaF + ACP), experimental nano-bioactive glass (6% n-BAG), and experimental nano-hydroxyapatite (10% n-HAP).

**Methods:**

The twenty-eight extracted primary molars were randomly divided into four groups: Group 1 (Enamel Pro), Group 2 (n-BAG), Group 3 (n-HAP), and Group 4 (control). Specimens were submerged in a demineralization medium for 96 h to create artificial enamel lesions measuring 4 × 4 mm. Commercial and experimental varnishes were applied to specimens, followed by 24 h of storage in artificial saliva and 7 days of pH cycling. The Scanning Electron Microscope and Energy Dispersive X-ray (SEM-EDX) were employed to assess the specimens at baseline, post-demineralization, post-remineralization, and following pH cycling. Lesion depth was examined by polarized light microscopy (PLM). Statistical evaluation was conducted using Repeated Measures ANOVA, Bonferroni post-hoc, Friedman, Wilcoxon signed-rank one-way ANOVA, Tukey’s post hoc, Kruskal-Wallis, Mann-Whitney, and Pearson correlation tests.

**Results:**

EDX showed highly significant intra-group increases in Ca Wt.% for all materials (*P* < 0.001). Inter-group differences were significant (*P* < 0.05) after remineralization and pH cycling. After both stages, the Ca/P ratio showed highly significant intra-group changes (*P* < 0.001) and significant inter-group differences. n-BAG recorded the highest Ca and Ca/P values, while the control showed the lowest. SEM images revealed complete and uniform surface coverage in n-BAG, partial surface recovery in Enamel Pro and n-HAP, and persistent porosities in the control. PLM confirmed the lowest lesion depth in n-BAG. A moderate negative correlation was found between lesion depth and the Ca/P ratio.

**Conclusion:**

n-BAG and n-HAP exhibited superior efficiency after pH cycling compared to Enamel Pro, suggesting their potential as preventive agents for managing initial enamel caries in primary teeth.

**Supplementary Information:**

The online version contains supplementary material available at 10.1186/s12903-026-08279-6.

## Background

Dental caries is regarded as one of the most widespread, chronic, multifactorial, transmissible infectious diseases worldwide, particularly in children. It occurs when the demineralization-remineralization balance is disturbed, leading to the loss of calcium and phosphate. Despite a decline in prevalence due to preventive strategies, dental caries remains a critical community health issue [1].

White spot lesions are the earliest indicator of tooth decay, presenting clinically as a dull whitish discoloration on the tooth surface. It exhibits subsurface mineral loss, whereas the superficial enamel layer usually remains intact, without cavitation, and can be reversed and restored through remineralization [2].

Saliva is essential for enamel remineralization, providing a supersaturated environment of fluoride, calcium, and phosphate ions that promotes mineral redeposition and buffers acidic challenges. This continuous ion availability supports the repair of early enamel lesions and modulates fluoride interactions and mineral deposition at the tooth surface [3].

Sodium fluoride (NaF) remains a gold-standard agent in caries prevention due to its proven remineralizing potential [4]. The 5% NaF varnish (22,600 ppm fluoride) provides sustained fluoride release at the enamel surface, demonstrating safety and effectiveness in promoting remineralization of early enamel lesions. Its mechanism involves the formation of a calcium fluoride–like reservoir and the promotion of fluorapatite formation, thereby increasing enamel resistance to repeated acid challenges [5, 6].

Recent research indicates diminished fluoride efficacy, as rapid fluorapatite deposition on the enamel surface creates a highly mineralized outer layer that acts as a diffusion barrier, restricting the penetration of calcium and phosphate ions into the deeper parts of the lesion. Consequently, remineralization may remain confined to the enamel surface, leaving the subsurface lesion inadequately repaired [7, 8]. Consequently, recent cariology research has focused on bioactive and biomimetic remineralizing materials that actively promote mineral deposition and structural repair of early enamel lesions [9–12].

The innovative enamel regeneration approach, including nano-hydroxyapatite materials and fluoride booster systems such as amorphous calcium phosphate and calcium sodium phosphosilicate (bioactive glass), has been utilized in the present study materials [13].

NaF combined with amorphous calcium phosphate (ACP) (Enamel Pro^®^ varnish) is incorporated as a varnish formulation. Upon application, ACP complex precipitates on the enamel surface and subsequently transforms into a stable hydroxyapatite phase, releasing calcium and phosphate ions that promote remineralization of the enamel subsurface lesion [14]. Recently, nanomaterials have been effective in remineralizing early carious lesions due to their high surface area, bioactivity, and biocompatibility [9, 15].

Recent advances in biomaterials have highlighted bioactive glass (BAG) as a multifunctional approach for caries management [16]. BAG consists of sodium–calcium phosphosilicate and interacts with body fluids to form hydroxycarbonate apatite (HCA), closely mimicking the chemical and structural features of natural tooth mineral [16, 17]. Through alkaline ion release, it exerts a pH-buffering effect that supports remineralization. At the same time, nanosized particles provide greater reactivity and deeper penetration into subsurface lesions than conventional fluoride- or calcium-based systems [18, 19]. In addition to enamel remineralization, BAG exhibits strong bioactivity and biocompatibility. It has shown effectiveness in bone regeneration, management of dentin hypersensitivity, antimicrobial activity against oral pathogens, and minimal cytotoxicity on pulp cells [16, 20]. Recent studies have demonstrated that BAG exhibits superior remineralization performance compared with conventional topical remineralizing agents [11, 21, 22].

Similarly, nano-hydroxyapatite (n-HAP), a biomimetic material structurally analogous to enamel crystals, promotes remineralization through direct mineral replacement [23]. Its nanoparticles can infiltrate enamel microporosities and serve as scaffolds for calcium and phosphate deposition, forming a new apatite layer [24]. n-HAP contributes to structural repair by restoring lost mineral content [25]. It also improves enamel microhardness, occludes dentinal tubules, and supports remineralization under acidic conditions by maintaining mineral supersaturation [25, 26]. Both in vitro and in vivo studies have demonstrated remineralization outcomes comparable to or exceeding those of conventional fluoride therapies [7, 27–30].

Gum rosin, also referred to as colophony, is a natural, renewable resin derived from the exudates of living pine trees. It possesses excellent film-forming properties, which have led to its widespread use as a carrier material in dental varnishes, including common fluoride-based formulations such as 5% sodium fluoride varnish (Duraphat) [31]. It serves as a safe, chemically stable, non-toxic, and biocompatible carrier for fluoride and other remineralizing agents [31, 32].

Numerous studies have used BAG and n-HAP materials as toothpaste. Unfortunately, this form lacks sufficient substantivity on the enamel surface and is filled with additives [33, 34]. Few studies have demonstrated that rosin-based varnishes containing bioactive components and peptides provide effective enamel remineralization while maintaining chemical stability and non-acidic properties [2, 4, 35]. Additionally, according to existing evidence, the remineralization potential of Enamel Pro varnish, n-BAG varnish, and n-HAP varnish in primary teeth has not been compared.

Therefore, the main purpose of the current research was to experimentally synthesize biomimetic materials, such as n-BAG and n-HAP, and incorporate them into hydrophobic rosin-based varnish formulations to evaluate their independent effects on remineralization efficiency compared with a commonly available NaF varnish. (Enamel Pro^®^).

## Methods

### Pilot study

A preliminary pilot study was implemented before the main investigation to refine procedures, assess its feasibility, and verify the reliability and consistency of the experimental procedures. Twelve primary enamel specimens (three from each group) were used to evaluate the demineralization solution and the creation of white spot lesions. The experimental varnishes were applied to evaluate the adhesion of rosin-based materials to the enamel surface. Scanning Electron Microscopy (SEM) verified the typical demineralization pattern (honeycomb structure), while Polarized Light Microscopy (PLM) on sectioned samples provided clear visualization of the lesion zones.

### Determination of sample size

A power analysis was conducted to verify sufficient power to test the null hypothesis that there is no difference among the tested groups. Also, it employed an alpha (α) level of 0.05, a beta (β) level of 0.05 (yielding a power of 95%), and an effect size (f) of 0.925, obtained from a previous study by Mashhour, A., et al. [36]. The required sample size (n) was set at 28. A sample size calculation was conducted utilizing R statistical analysis software, version 4.3.2, for Windows.

### The preparation of materials

#### Bioactive glass (45S5) Nanoparticles synthesis

A bioactive glass powder was synthesized utilizing the sol-gel method to minimize particle size and improve bioactivity [22]. Colloidal solutions with a composition of 45S5 were synthesized by combining deionized water, 2 N hydrochloric acid, tetraethyl orthosilicate (TEOS), triethyl phosphate (TEP), and calcium nitrate. The primary step is mixing TEOS with ethanol as a solvent. Deionized water was incorporated into the solution, and the preparation was agitated till a homogeneous solution was formed. The molar ratio of H₂O to TEOS was 4:1. After 30 min, TEP was added to the agitated solution. Calcium nitrate was subsequently introduced after another 30 min. The solution was then agitated for an extra hour. The resulting gel was subjected to heating (80 °C for 10 h), drying (140 °C for 15 h), and thermal stabilization (650 °C for 2 h) in accordance with a standardized protocol [37].

#### Hydroxyapatite nanoparticles synthesis

Hydroxyapatite nanoparticles were prepared by the wet-chemical approach described by Jarcho et al. [38] and Paz et al. [39]. A calcium chloride aqueous solution was intensely agitated at room temperature. Ammonium hydrogen phosphate solution was gradually introduced drop by drop into the calcium chloride solution. Ammonia solution was introduced drop by drop to reach a pH of 10. After maintaining the reaction at 40 ˚C for 4 h, it was agitated at room temperature for an additional sixteen hours. A white crystalline form of hydroxyapatite was obtained.

$$10\mathrm{Ca}\;(\mathrm{OH})2\:+\:6\mathrm H3\mathrm{PO}4\;\rightarrow\;\mathrm{Ca}10(\mathrm{PO}4)6(\mathrm{OH})2\:+\:18\mathrm H2\mathrm O$$

### Varnishes preparation

#### Preparation of n-BAG varnish

Gum rosin (colophony) was used as the varnish matrix due to its application in dental varnish formulations as a biocompatible film-forming carrier that enhances enamel adhesion and supports sustained ion release. Rosin-based resins are commonly incorporated into commercially available fluoride varnishes, such as 5% sodium fluoride varnish formulations (Duraphat), supporting their safety and applicability in dental remineralization studies [31]. It was utilized to produce the BAG varnish. Solid colophony was crushed into small particles, dissolved in ethanol (Chem-Lab, Belgium), and added to a 40% w/v concentration through heating at 60 ˚C with vigorous agitation. After that, bioactive glass was added to the previous solution at 6% w/v, and the formulation was agitated for 1 h to obtain a clear solution. Finally, Hydroxypropyl methylcellulose (HPMC) 20–40% w/v was used as a gelling agent to increase varnish viscosity [40, 41].

#### Preparation of n-HAP varnish

As in the previous method, a homogeneous solution was obtained by adding HAP at 10% w/v and stirring for 1 h. Finally, Hydroxypropyl methylcellulose (HPMC) 20–40% w/v was used as a gelling agent to increase the varnish viscosity [41, 42].

#### Preparation of placebo varnish

According to the previously described method, a placebo varnish was developed using blank rosin, devoid of active ingredients.

### Characterization of varnishes [9, 22]

The surface morphology and particle sizes of nano-bioactive glass and nano-hydroxyapatite were examined utilizing transmission electron microscopy (TEM) and X-ray diffraction (XRD) to evaluate their crystalline nature.

**Fig. 1:**
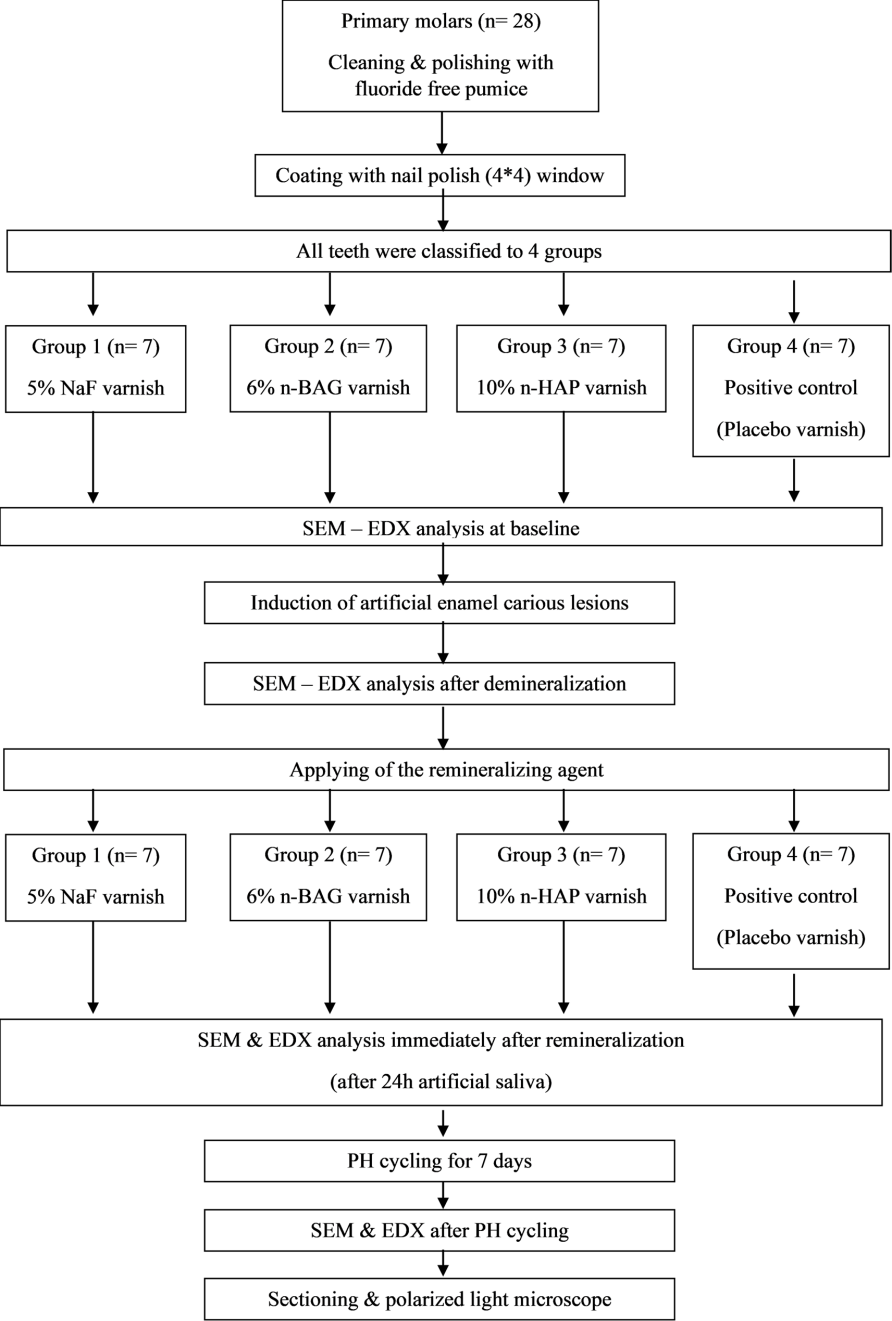
Workflow of the study methodology

The workflow of the study methodology is shown in Fig. 1.

### Sample preparation procedure

Twenty-eight human primary molars, either extracted for orthodontic purposes or normally exfoliated, were involved. All teeth were polished using a non-fluoride slurry under water irrigation to obtain a clean enamel surface [36, 40]. The teeth were gently dried and meticulously inspected. The examination was conducted visually with the naked eye, using a 3x magnifying lens. Teeth exhibiting evident structural defects, cavities, initial caries lesions, or discolored teeth were excluded. Following extraction, all teeth were cleaned of debris and stored in 0.1% thymol solution to prevent microbial growth. The samples were preserved in sealed, light-resistant containers at room temperature in the Pediatric Dentistry Department laboratory and used within one month post-extraction [43]. In the current study, second primary molars were selected because of their relatively larger crown dimensions and broader, flatter buccal surfaces compared with first primary molars, which allow adequate accommodation of a standardized 4 × 4 mm window. On the buccal surface, the center region of the middle third of each tooth was isolated with a 4 × 4 mm window of adhesive label, which was subsequently covered with two coats of acid-resistant nail varnish (Amanda A.R.E.). After drying, the adhesive labels were removed, exposing a standardized 4 × 4 mm window on the enamel surface [36, 40]. After that, baseline mineral content and morphology were evaluated using SEM-EDX [44].

### Artificial carious lesions induction

Artificial enamel lesions were induced by individually submerging each specimen in 10 mL of a demineralizing solution (Table 1) for 4 days, with the solution being renewed daily. The required components were dissolved in distilled water under continuous magnetic stirring to ensure complete dissolution and homogeneity. The acidity of the solution was then adjusted to the target pH of 4.4. The final pH was confirmed using a calibrated digital pH meter before application. After preparation, the solution should be stored in a tightly sealed container to prevent contamination, evaporation, and pH changes, thereby maintaining stable experimental conditions [36]. After preparation, the solution should be stored in a tightly sealed container to prevent contamination, evaporation, and pH changes, thereby maintaining stable experimental conditions. The procedure was maintained until noticeable alterations were observed in the tooth enamel under dry and wet circumstances, corresponding to an ICDAS score of 2 [45]. Formation of white-spot lesions was then assessed using SEM and EDX [44].

**Table 1:** Materials utilized in this research

Material	Composition	Manufacturer
5% sodium fluoride varnish (Enamel pro varnish)	5% Sodium fluoride& varnish 2.26% Amorphous Calcium Phosphate (Rosin, ethanol, 5% sodium fluoride, dibasic sodium phosphate, calcium sulfate dihydrate)	(Premier Dental Products Co., USA)
6% n-BAG containing varnish (Experimentally manufactured)	45% SiO2, 24.5% CaO, 24.5% Na2O and 6% P2O5 and varnish.	(prepared by Nano Gate Company, Cairo, Egypt)
10% n-HAP-containing varnish (Experimentally manufactured)	39.68% by weight calcium and 18% by weight phosphorus, resulting in a Ca/P mole ratio of 1.67 and varnish.	(Synthesized by Nano Gate Company, Cairo, Egypt)
Placebo varnish	35–40% colophony(rosin), 10–30% ethanol,20–40% Hydroxypropyl methylcellulose (HPMC)	(Synthesized by Nano Gate Company, Cairo, Egypt)
Demineralization solution	(2.20 mmol/L calcium chloride, 2.20mmol/Lmonosodium_ phosphate, 1 mol/L potassium hydroxide, and 0.05 mol/L acetic acid with pH 4.4)	Laboratory prepared in the Faculty of Science, Al Azhar University
Remineralization solution	(1.5mmol/L calcium chloride, 0.9 mmol/L monosodium- phosphate, 150 mmol/L potassium chloride with pH 7.0)	Laboratory prepared in the Faculty of Science, Al Azhar University.
Artificial saliva	3.9 mM Na3 PO4, 4.29 mM NaCl, 17.98 mM KCl, 1.1 mM CaCl2, 0.08 mM MgC12,0.5 mM H2S04, 3.27 mM NaHCO3 with distilled water	Laboratory prepared in the Faculty of Science, Al Azhar University.

### Grouping of specimens and application of remineralizing agents

After forming white spot lesions, each specimen was meticulously washed with deionized water for 1 min and then reinserted into its test tube. Specimens were randomly divided into four groups(*n* = 7) and labeled as G1, G2, G3, and G4:

- Group 1: Sodium fluoride varnish (5%) + amorphous calcium phosphate (ACP) (Enamel Pro)
- Group 2: Nano-bioactive glass varnish (6%) (n-BAG)
- Group 3: Nano-hydroxyapatite varnish (10%) (n-HAP)
- Group 4: Control group (Placebo varnish)

To ensure standardization, all applications were performed by the same operator under identical experimental conditions using a uniform application thickness across all groups.

In Group 1, the enamel surfaces were cleaned with deionized water and air-dried with compressed air before a thin layer of 5% sodium fluoride varnish (Enamel Pro^®^) was applied using the applicators supplied with the commercial product. The samples were allowed to air-dry for 5 min and were then stored in artificial saliva for 24 h. The varnish was carefully removed using a cotton swab saturated with acetone to avoid damage to the enamel surface, followed by rinsing with deionized water for 1 min prior to SEM-EDX evaluation [36, 44]. The removal was essential because, unlike in vivo conditions, where salivary flow, chewing, brushing, tongue movement, and oral clearance naturally remove varnish over time, in vitro conditions do not replicate these dynamic processes. Removing the varnish allowed accurate assessment of the enamel surface morphology and the effects of released ions, reflecting true remineralization activity. This approach is consistent with previous in vitro remineralization studies, where varnish removal is performed to quantify enamel surface recovery and ion incorporation [36, 46].

For Groups 2 (n-BAG glass), 3 (n-HAP), and 4 (placebo varnish), the experimental materials were applied using a microbrush. All subsequent procedures were performed identically to those described for Group 1.

### The pH Cycling

All groups were treated with a 7-day pH cycling protocol, consisting of daily immersion of each specimen in 10 mL of demineralizing solution (4 h) followed by 10 mL of remineralizing solution (20 h), refreshed daily. The remineralizing solution (Table 1) was prepared using analytical-grade reagents, sequentially dissolved in deionized water under continuous magnetic stirring to ensure complete homogenization. The solution pH was carefully adjusted to 7.0 and verified with a calibrated digital pH meter. All prepared solutions were stored in sealed, light-resistant containers at room temperature to maintain chemical stability and prevent potential degradation. The samples were assessed using SEM-EDX after the PH cycle [44, 46]. 

### Evaluation of samples

Single-blinded examiners evaluated SEM-EDX and PLM results to avoid operator bias.

#### SEM-EDX Analysis [40, 44]

Energy-dispersive X-ray spectrometry (EDX) (Inspect S, FEI Company, Netherlands) was combined with a scanning electron microscope (FEI Company, Netherlands) and employed as a semi-quantitative, semi-qualitative analytical technique to assess the mineral content of the enamel and examine the surface morphology of the samples at baseline, post-demineralization, post-remineralization, and following pH cycling. SEM–EDX analyses were performed in low-vacuum (variable pressure) mode, which allowed examination of enamel specimens without applying conductive coating, thereby preserving the native surface chemistry and preventing interference with subsequent remineralization procedures and PLM analysis. Specimens were gently removed from deionized water and allowed to air dry at room temperature inside the SEM antechamber prior to analysis, without the use of absorbent paper or any material that could cause surface contamination or mechanical disruption. Imaging time was minimized and standardized across all experimental stages (accelerating voltage: 20 kV) to limit cumulative electron beam exposure. Immediately after each SEM–EDX session, specimens were transferred into individually labeled conical centrifuge tubes containing fresh deionized water and placed in tube racks to ensure complete immersion and maintain specimen hydration between experimental stages. The expected pattern of elemental changes across the four experimental stages confirmed that the analytical procedure did not adversely affect the specimen [47–49]. 

The samples were examined under SEM at magnifications of 500x, 1000x, and 2000x. The distribution of calcium (Ca), phosphorus (P), and fluoride (F) elements on the enamel surface was calculated as weight% and represented as peaks on a graph, with their corresponding measurements. Each Ca and P content group was converted to a Ca/P ratio.

#### Lesion depth evaluation: by polarized light microscope (PLM)

All SEM imaging and pH cycling procedures were carried out on intact enamel specimens without prior sectioning to preserve the original surface morphology. After completion of the final SEM imaging and pH cycling, the specimens were rinsed with deionized water and prepared for qualitative analysis using polarized light microscopy. The enamel corresponding to the previously defined standardized 4 × 4 window was longitudinally sectioned into two halves using a low-speed diamond disc under continuous water cooling. One-half was randomly selected by coin toss and ground using silicon carbide paper to obtain thin longitudinal sections (approximately 100–150 μm) for PLM evaluation.

This sequence ensured that sectioning was performed only after all surface analyses and was confined to the same region of interest, allowing accurate correlation between SEM and PLM findings. The microscope (DM LM, Leica Microsystems, Wetzlar, Germany) was equipped with a Leica digital camera and operated through the Leica Application Suite (LAS) software (Faculty of Dentistry, Ain Shams University) [36].

Images were captured and analyzed directly under the microscope at a magnification of 25×, using a 2.5× objective lens and a 10× eyepiece to quantify the subsurface depth of the lesion body that represented the greatest demineralization area [50, 51]. The deepest demineralized area was quantified (µm) at three specific areas(the center of the lesion, the deepest portion as well as its peripheries) within the subsurface of the lesion body, oriented vertically to the superficial enamel surface and reaching to the translucent zone, with the mean value determined and documented as the lesion depth of the specimens [50, 52].

### Statistical analysis

The SPSS statistical package (version 25, IBM Co., USA) was employed to conduct the statistical analysis. For intragroup comparisons (comparing Baseline, Demin, Remin, and pH cycling stages within the same group), Repeated Measures ANOVA was applied for normally distributed data, followed by Bonferroni post-hoc correction for pairwise comparisons between stages. Mauchly’s test of sphericity was conducted, and when violated (*p* < 0.05), the Greenhouse-Geisser correction was applied. For non-normally distributed data (F Wt.%), the Friedman test (non-parametric equivalent of Repeated Measures ANOVA) was used, followed by the Wilcoxon signed-rank test with Bonferroni correction for pairwise comparisons. For intergroup comparisons (comparing the four groups at each specific stage), One-Way ANOVA was applied for normally distributed data, followed by Tukey’s HSD post-hoc test for pairwise comparisons between groups. For non-normally distributed data, the Kruskal-Wallis test was used, followed by the Mann-Whitney U test with Bonferroni correction for pairwise comparisons between groups. Pearson’s Correlation Coefficient was applied to examine the correlation between Ca/P ratio and lesion depth results. Pearson’s correlation is a parametric statistical test used to assess the intensity and direction of a linear relationship between two variables. Its value ranges from − 1 (strong negative relationship) to + 1 (strong positive relationship). P-value ≤ 0.05 was considered statistically significant (95% significance level). P-value ≤ 0.001 was considered highly statistically significant (99% significance level). Data normality was evaluated using the Shapiro–Wilk test.

## Results

All experimental groups, except the control group, demonstrated an improvement in mineral gain following remineralization. The n-BAG group exhibited the most pronounced enhancement, reflected by higher Ca/P ratio values and reduced lesion depth compared with the other tested group.

To enhance readability and simplify data interpretation, statistical analysis focuses on Ca/P ratio and lesion depth, while detailed elemental wt% data are provided in the Supplementary Material.

### I-EDX analysis

#### Ca/P ratio

**Figure** 2

(Intra-group comparison) For Enamel Pro and n-BAG, no significant differences were found between the baseline and remineralization stages. In contrast, significant differences were identified between the demineralization stage and the other three stages, as well as between the pH and the other three stages. For the n-HAP group, significant differences were recorded between the baseline and the other stages, as well as between demineralization and the other three stages. In contrast, no significant difference was detected between remineralization and the pH stages. For the control group, significant differences were detected between the baseline and the other stages, as well as between the after-pH cycling stage and the other three stages. However, no significant difference was found between the demineralization and remineralization stages.

**Fig. 2:**
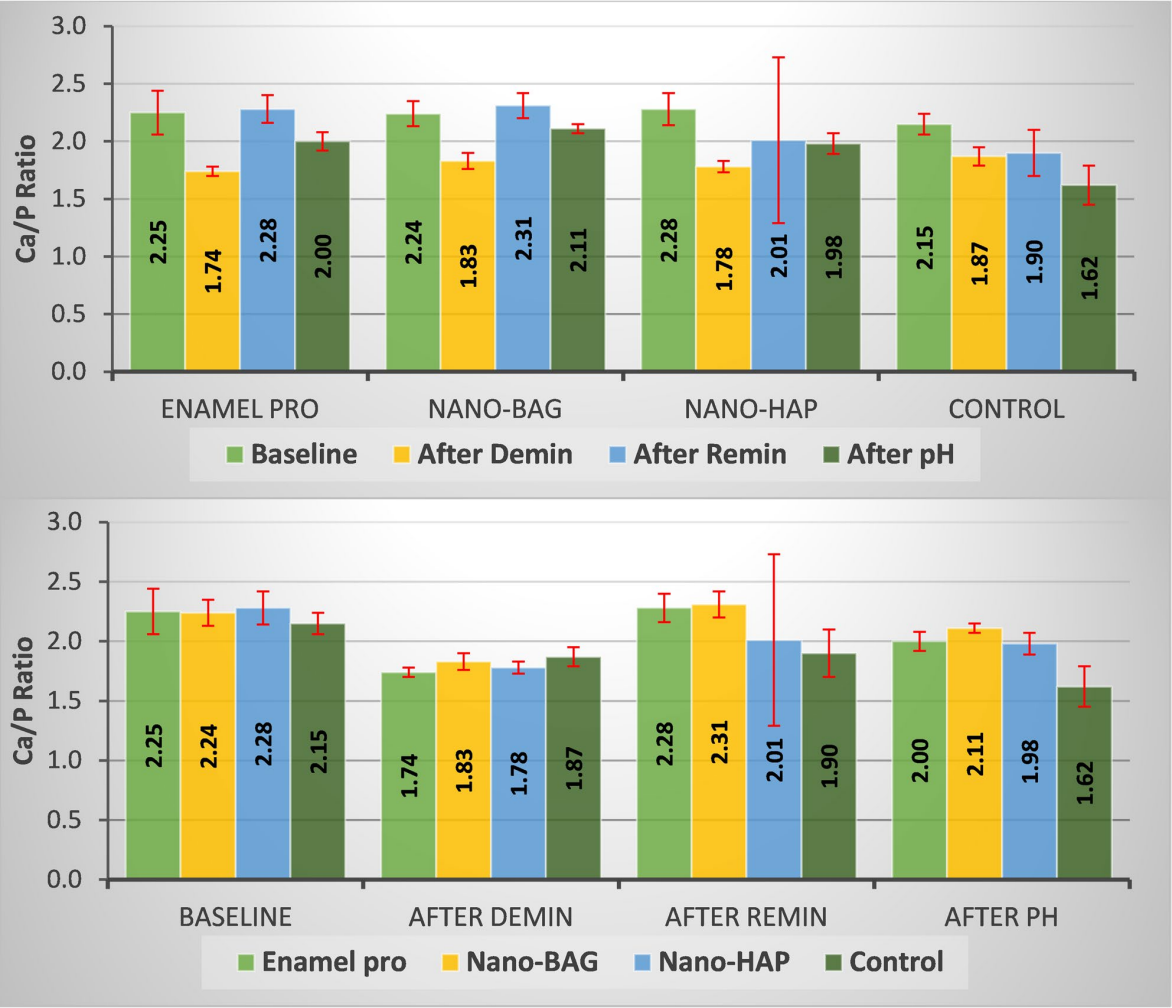
A bar chart representing mean ± SD for intra- and Inter-group comparison of Ca/P ratio

The Repeated Measures ANOVA results revealed that the overall P value for intra-group comparisons was statistically highly significant (*P* < 0.001) in the Enamel Pro, n-BAG, and control groups. The n-HAP group demonstrated statistically significant differences (*p* < 0.05). The significance lies mainly in the highest Ca/P ratio at the remineralization stage and the lowest at the demineralization stage (Table 2).

**Table 2:** Mean ± SD, intra, and inter-group comparison of Ca/P ratio

	Baseline	After demin	After remin	After pH	*P*-value*
Enamel pro	2.25 ± 0.19^Aa^	1.74 ± 0.04^Ac^	2.28 ± 0.12^Aa^	2 ± 0.08^Ab^	< 0.001^HS^
n-BAG	2.24 ± 0.11^Aa^	1.83 ± 0.07^Ac^	2.31 ± 0.11^Aa^	2.11 ± 0.04^Ab^	< 0.001^HS^
n-HAP	2.28 ± 0.14^Aa^	1.78 ± 0.05^Ac^	2.01 ± 0.72^ABb^	1.98 ± 0.09^Ab^	0.018^S^
Control	2.15 ± 0.09^Aa^	1.87 ± 0.08^Ab^	1.90 ± 0.2^Bb^	1.62 ± 0.17^Bc^	< 0.001^HS^
*P*-value**	0.167^NS^	0.319^NS^	0.045^S^	< 0.001^HS^	

Small letters for pairwise comparison between different time intervals, while the capital letters are for pairwise comparison between groups. There is no significant difference between means that share at least one superscript letter at a significance level of *P* ≤ 0.05

* Overall P-value for Intra-group comparison between the four time intervals (Repeated Measures ANOVA test)

** Overall P-value for Inter-group comparison between the four groups (ANOVA test)

S Statistically significant at *P* ≤ 0.05, NS Non-significant *P* < 0.05

HS Highly significant at *P* ≤ 0.001

##### (Inter-group comparison)

The n-BAG group had the highest mean Ca/P ratio after the remineralization stage, compared to the other three groups. In comparison, the control group had the lowest mean at the same stage.

The ANOVA results indicated a significant inter-group difference (*P* < 0.05) after the remineralization stage, a highly significant difference (*P* < 0.001) after the pH cycling stage, and a non-significant difference (*P* > 0.05) at baseline and after the demineralization stages.

### II-SEM results

**Fig. 3:**
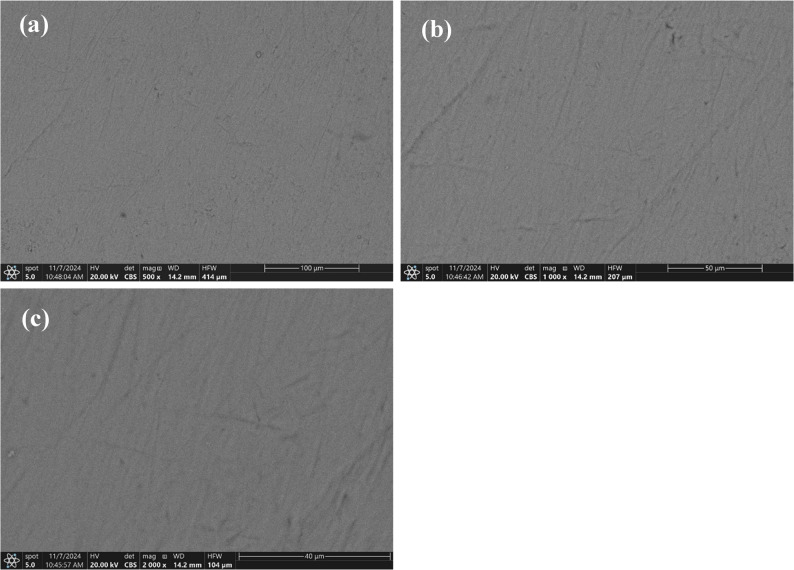
SEM images of enamel specimen at baseline. **a** 500×, (**b**) 1000×, and (**c**) 2000× magnifications, showing the typical morphology of sound, intact enamel

**Fig. 4:**
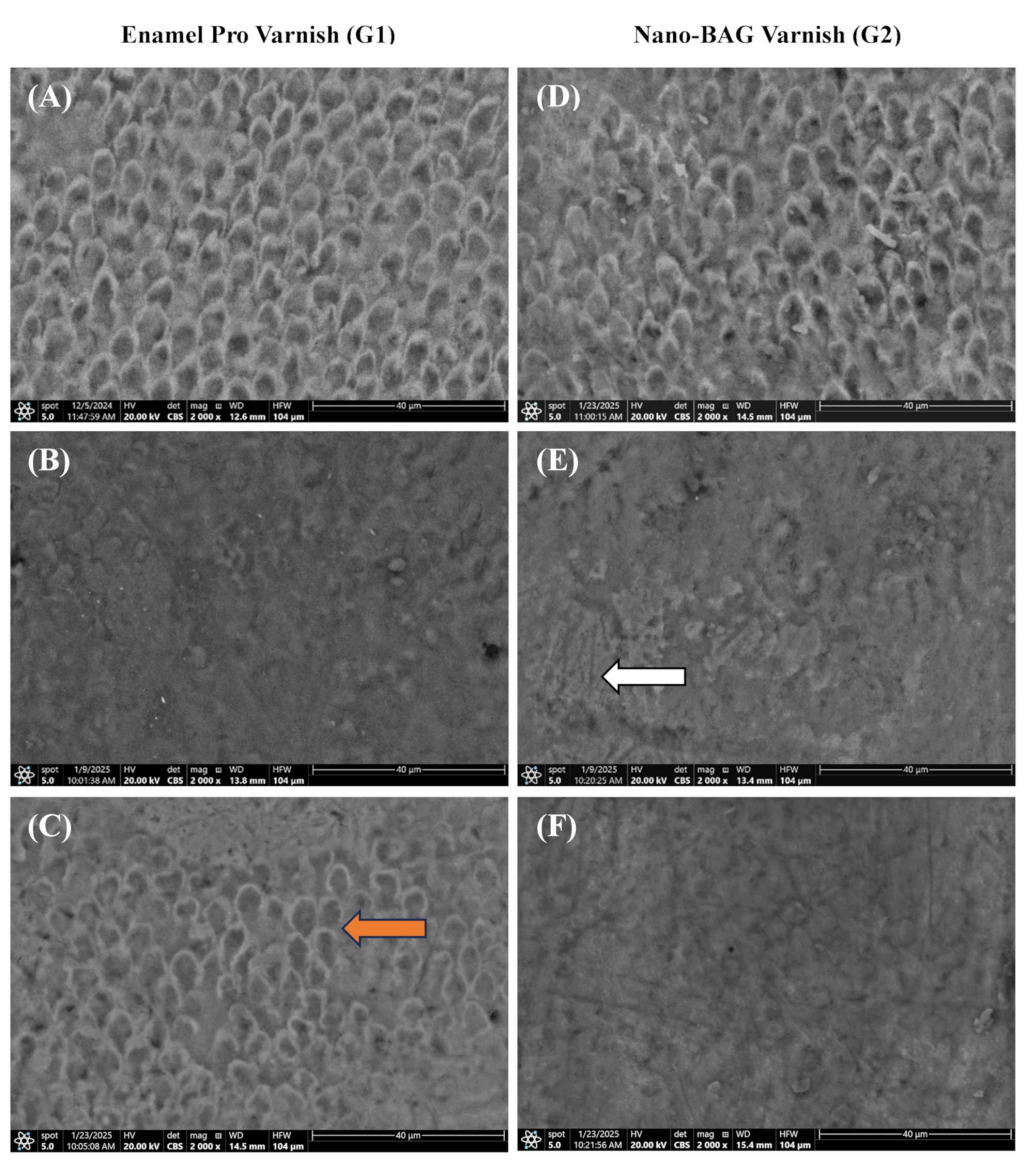
SEM images of enamel specimens. **A**,** D** After demineralization in groups 1 and 2, the samples exhibited a characteristic honeycomb (keyhole) structure and a demineralized enamel surface pattern, with loss of the prism core structure. **B** After remineralization in group 1 at 2000× magnification, a precipitation layer covering the enamel surface and a pronounced reorganization of surface morphology were observed. **C** Following pH cycling in group 1, partial crystalline deposition, accompanied by a mineral recovery pattern, was evident on the enamel surface. The reappearance of the keyhole, along with exposed enamel prisms, could be detected in certain areas (orange arrow). **E** After remineralization in group 2 at 2000× magnification, a uniform coverage of the enamel surface with organized crystals was observed; the honeycomb pattern disappeared, and homogeneous hydroxyapatite deposition on the enamel surface was evident (white arrow). **F** After pH cycling in group 2, the enamel surface showed the most dense and integrated morphology, with a persistent covering layer and no visible keyhole pattern

**Fig. 5:**
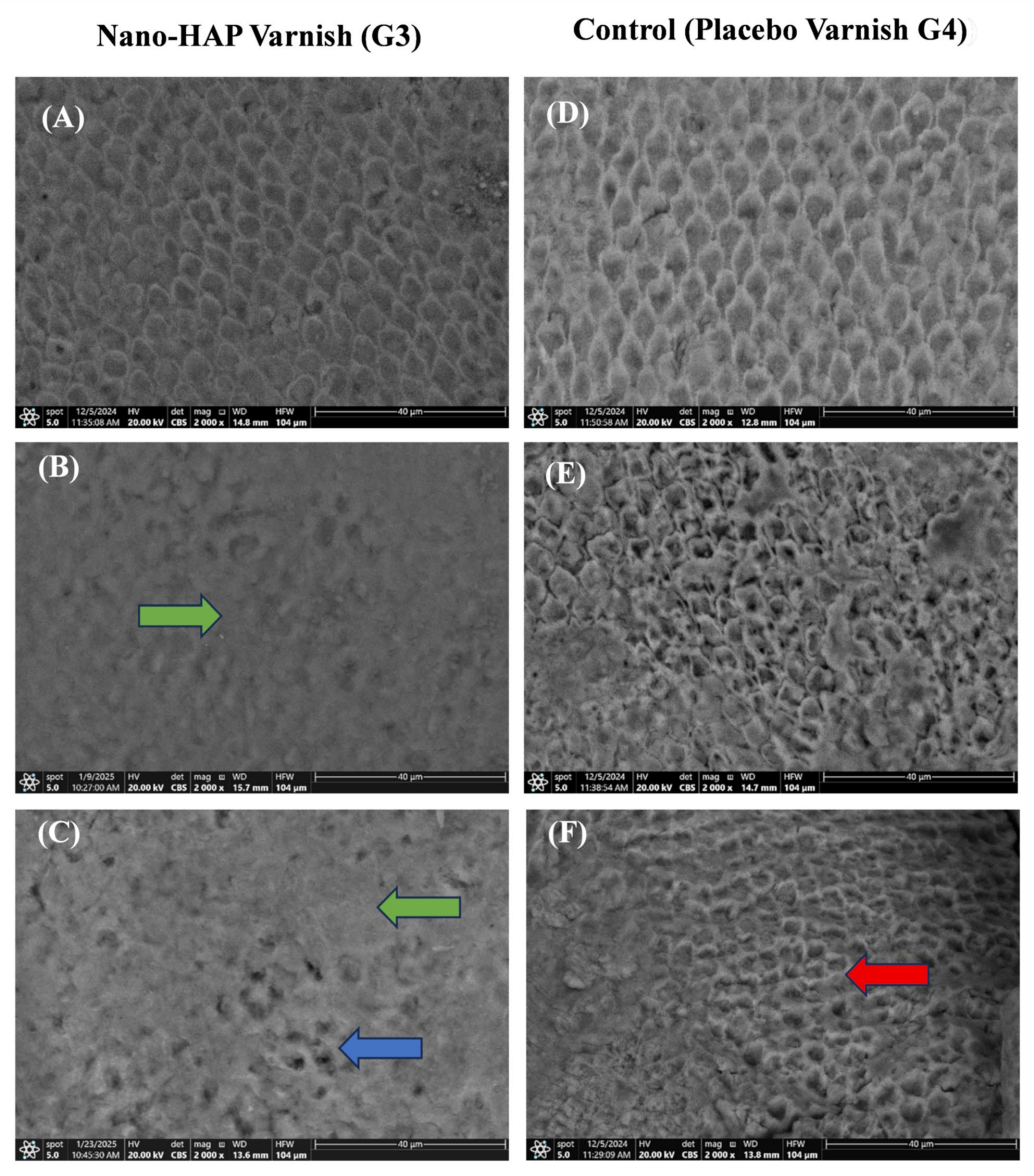
SEM images of enamel specimens. **A**,** D** After demineralization in groups 3 and 4, the samples exhibited a characteristic honeycomb (keyhole) structure and a demineralized enamel surface pattern, with loss of the prism core structure. **B** After remineralization in group 3 at 2000× magnification, homogeneous deposition of nano-HAP crystals on the enamel surface (green arrow) was observed. **C** Following pH cycling in group 3, stable crystal adhesion was observed. Slight microporosities were also observed (blue arrow). After remineralization in group 4 (**E**) at 2000× magnification, surface destruction and noticeable porosity were evident. **F** After pH cycling in group 4, irregular and deep porosities were observed in the demineralized enamel (red arrow)

Images of the enamel specimen at baseline, after demineralization, after remineralization, and following the pH cycle are shown in Figs. 3, 4, and 5.

### III-polarized light microscope (PLM) results

**Fig. 6:**
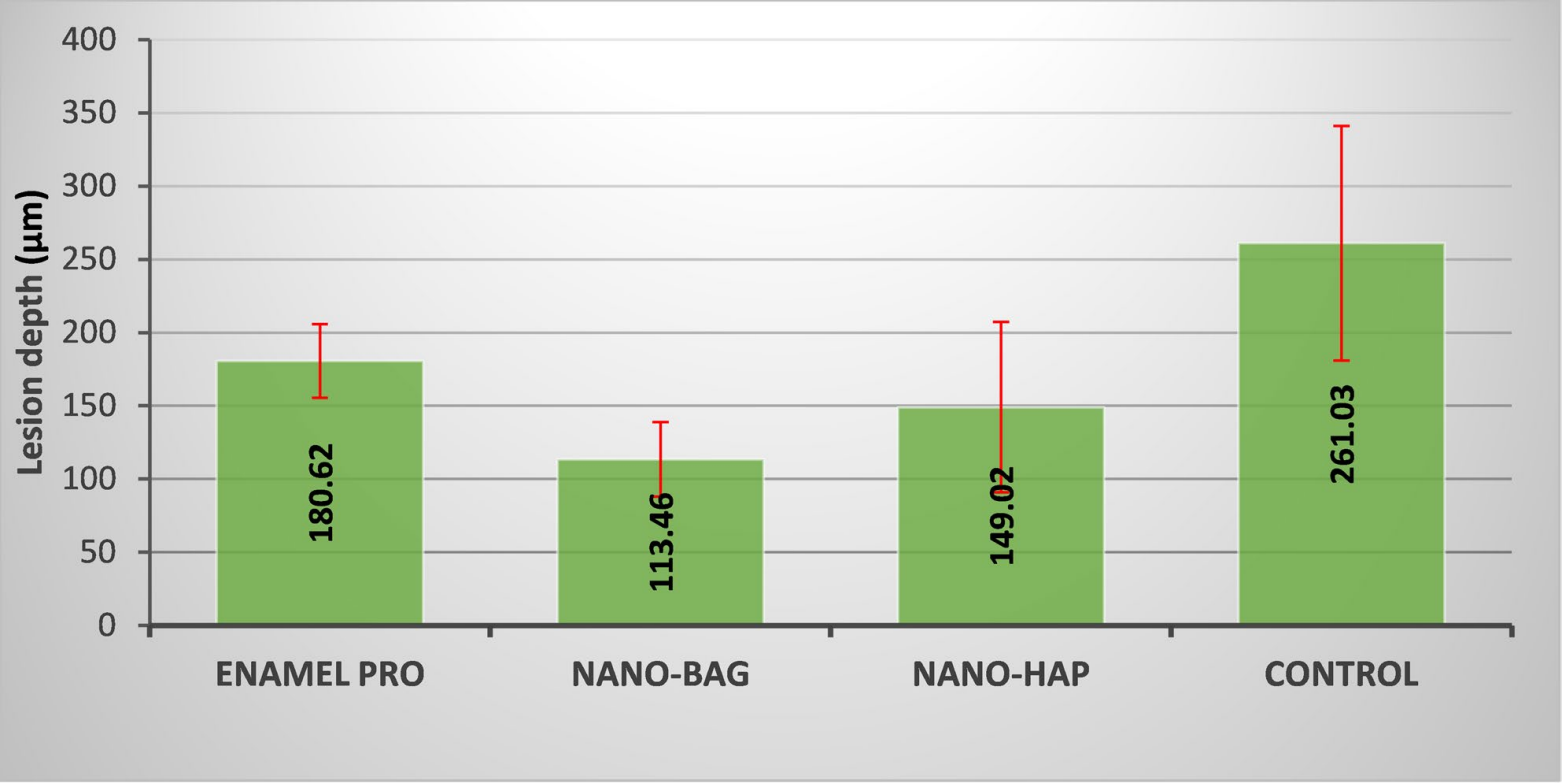
A bar chart representing the mean and SD of lesion depth for the studied groups

In Fig. 6, no significant differences in mean lesion depth were found between the Enamel pro and n-HAP groups, nor between the n-HAP and n-BAG groups, as confirmed by the Tukey Post Hoc pairwise comparison. In contrast, significant differences were revealed between the n-BAG and Enamel Pro groups, as well as between the n-BAG and control groups. ANOVA test showed a highly significant overall intergroup difference (*P* < 0.001). This significance is primarily due to the difference in mean lesion depth between the highest (control group) and the lowest (n-BAG group) (Table 3).

**Table 3:** Mean, SD, and inter-group comparison of lesion depth (µm) for the studied groups

	Mean	SD
Enamel Pro	180.62^B^	25.08
n-BAG	113.46^C^	25.53
n-HAP	149.02^BC^	58.12
control	261.03^A^	80.12
P-value**	< 0.001^HS^	

There is no significant difference between means that share at least one superscript letter at a significance level of *P* ≤ 0.05

*HS* Highly significant at *P* ≤ 0.001

### Correlation between the EDX results (Ca/P ratio) and the lesion depth results

**Fig. 7:**
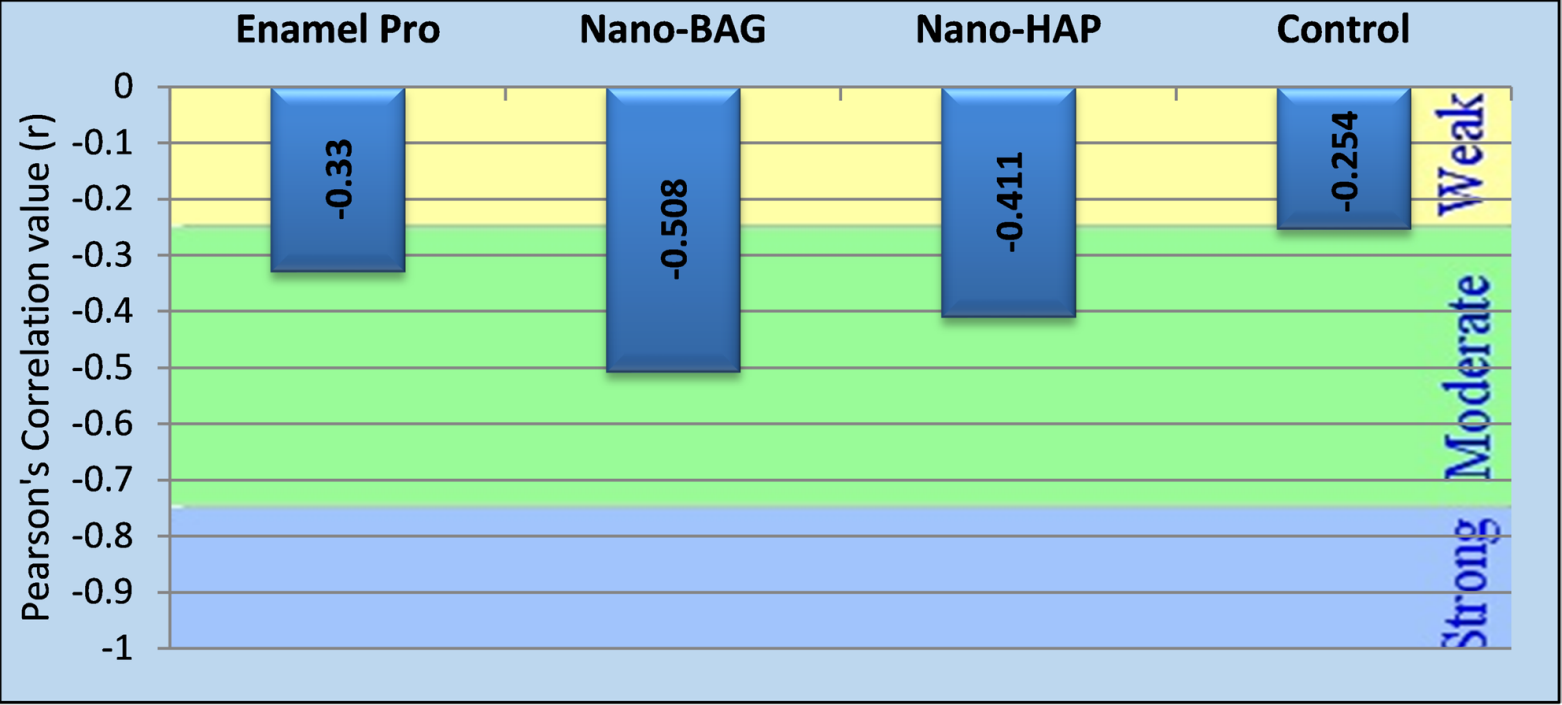
Bar chart showing the Correlation value (r) between Ca/P ratio and lesion depth

In Fig. 7, the Enamel Pro group revealed a correlation coefficient of -0.330. In contrast, n-BAG (*r* = -0.508) and n-HAP (*r* = -0.411) showed stronger associations, while the control group showed the weakest correlation (*r* = -0.254). Statistical significance was found in all groups (*p* < 0.05 for Enamel Pro, n-HAP, and control; *p* < 0.001 for n-BAG), confirming that increased Ca/P ratios consistently decreased lesion depth. Finally, the strength of this association differed moderately among treatment groups (Table 4).

**Table 4:** Correlation between Ca/P ratio and lesion depth results in the different studied groups

Group	r**	*P*-value	Correlation type
Enamel Pro	− 0.330	0.011^S^	Moderate Negative
Nano-BAG	− 0.508	< 0.001^HS^	Moderate Negative
Nano-HAP	− 0.411	0.007^S^	Moderate Negative
Control	− 0.254	0.046^S^	Moderate Negative

S Significant (Correlation is significant at the 0.05 level). - HS: Highly significant (Correlation is significant at the 0.01 level)

**Correlation value from Pearson’s Correlation test, to measure the strength and direction of the linear relationship between Ca/P ratio results and lesion depth results.

## Discussion

The remineralization of early carious lesions constitutes an essential aim of modern non-invasive dentistry [53]. Recently, nanomaterials have been widely used in dentistry as alternatives to fluoride due to their improved characteristics, including a greater surface area [54]. Numerous studies have demonstrated the efficacy of toothpaste containing bioactive and nano-hydroxyapatite in comparison to other toothpastes containing fluoride and calcium phosphate in terms of microhardness, SEM-EDX, and PLM [7, 29, 55, 56].

The rationale for selecting a varnish formulation rather than a gel or toothpaste was based on clinical applicability in very young children. Gel or toothpaste formulations typically require greater patient cooperation, controlled application time, and, in some cases, the use of trays and suction, which may not be feasible in children aged 2–3 years, particularly those presenting with early white spot lesions [57]. In contrast. The colophony (rosin)-based varnish used in this study allows rapid chairside application, minimal dependence on compliance, and prolonged enamel adherence. making it more clinically applicable in pediatric patients under six years of age [31, 32]. Colophony, when ground and dissolved in ethanol and coated onto the enamel surface, sets and forms a substance with the consistency of gel upon the evaporation of the alcohol [41, 58].

Several studies have confirmed that toothpastes containing 10% nano-hydroxyapatite are the optimal concentration for remineralizing initial carious lesions [49, 59]. R. Zhang (2021) conducted an in vitro study to examine the remineralization effectiveness of various concentrations of bioactive glass on artificial enamel lesions in primary teeth. The results revealed that 6% BAG exhibited the greatest mineral recovery and superior remineralization impact upon artificial carious lesions, as evidenced by SEM-EDX analysis [40].

That’s why in the current study, 6% n-BAG-containing varnish and 10% n-HAP-containing varnish were experimentally manufactured to evaluate the independent effects of synthetic remineralization agents on enamel surface remineralization and to compare their efficiency with that of a commonly available 5% NaF varnish. (Enamel Pro^®^ varnish).

Nano-bioactive glass was prepared using the sol-gel technique to obtain a small particle size and enhance bioactivity [37]. Hydroxyapatite nanoparticles were prepared using a wet chemical method [21] and characterized by transmission electron microscopy (TEM) and X-ray diffraction (XRD) to confirm the crystalline nature of the tested materials [9, 22].

A pH-cycling regime was used in this in vitro study to evaluate remineralization, as it mimics the oral cavity’s acid-challenge conditions [11, 29]. The pH cycling procedure employed in the current research was based on a principle developed by ten Cate and Duijsters [60], Thaveesangpanich et al. [61], who revealed that an in vitro pH-cycle procedure for 10 days induced carious lesions in deciduous teeth involving enamel and dentin, illustrating that 7 days is the optimum duration for the pH-cycle in the enamel surface of deciduous teeth. Consequently, the specimens utilized in this investigation underwent pH cycling for 7 days [44, 46].

In this study, all groups undergo SEM-EDX analysis at four stages: baseline, post-demineralization, post-remineralization, and after pH cycling. SEM/EDX analysis provides semi-quantitative elemental assessment to evaluate relative elemental variations among experimental groups and to support compositional assessment of enamel surfaces. Particular emphasis was placed on calculating the Ca/P ratio, which served as the primary indicator of mineral changes occurring during the different experimental phases. The relative elemental values obtained from EDX were therefore used to support the compositional interpretation of enamel surface changes, while polarized light microscopy offered complementary qualitative evaluation of sub-surface lesions. The Ca/P ratio was calculated for each group’s calcium and phosphate contents [49, 59, 62, 63].

The characteristics of biologically relevant calcium phosphates are significantly influenced by their (Ca/P) atomic ratios. The Ca/P ratio of normal enamel (Ca10(PO4)6(OH)2) was found to be 1.667 [64]. Consequently, the accuracy in determining this ratio is essential.

**Fig. 8:**
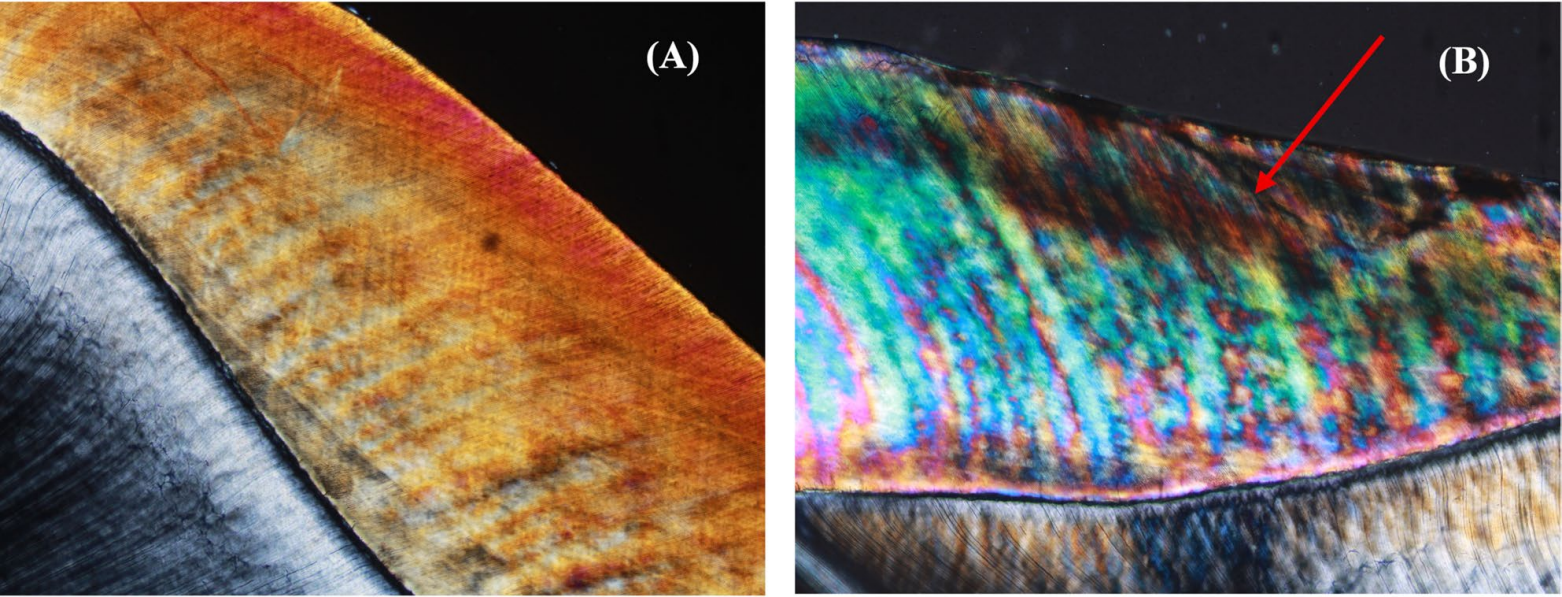
Polarized light micrographs of sectioned specimens. **A** Sound enamel showed typical surface remineralization and normal birefringence. **B** Enamel with a sub-surface lesion showed an evident demineralized enamel band with positive birefringence

The microscopic alterations observed in the carious lesion zones were evaluated qualitatively using a polarized light microscope, which is considered the most descriptive and sensitive histological method [50] (Fig. 8).

After the remineralization process, the surface layer exhibits a more pronounced negative birefringence, which suggests that mineral deposition in the area has increased. Conversely, a partial recovery of the mineral content was suggested by evidence of a decrease in positive birefringence within the lesion body. We also demonstrated a consistent moderate negative relationship between Ca/P ratios and lesion depth across all groups, supporting their remineralization potential.

This study revealed no significant differences among the four groups at baseline and following demineralization. It shows that standardized, homogeneous artificial enamel lesions were created utilizing the demineralization solution.

Following remineralization, the mean values of Ca, P, and F wt%, as well as the Ca/P, increased throughout the remineralization stage in all groups, excluding the control group, indicating the efficacy of the remineralization process. The n-BAG and the Enamel Pro groups demonstrated higher post-remineralization values than their demineralization levels. The n-BAG group demonstrated better performance in replenishing minerals lost during demineralization than the other three groups.

In this study, the improvement in remineralization by n-BAG varnish, as indicated by SEM-EDX results, may be attributed to the precipitation of crystallized hydroxycarbonate apatite resulting from the interaction of bioactive glass with saliva. This form showed higher stability than amorphous calcium phosphate, serving as a reservoir for releasing additional Ca and P ions [65].

The SEM results of n-BAG varnish revealed complete crystalline deposition and uniform surface coverage, aligning with the findings by Akbarzade, Tina, et al., who found that an SEM image of the CPP-ACP involving n-BAG revealed amorphous hydroxyapatite crystals covering the demineralized enamel structure [9] (Fig. 4).

This finding may be attributed to the mechanism of bioactive glass, which involves the release of Ca and P ions through the exchange of Na ions with hydrogen cations (H₃O⁺) in a moist environment. These reactions occur within seconds. The release of Ca and P ions continues as long as the material remains in a humid environment. The release of Na ions induces a localized, transient increase in pH, which facilitates the formation of an apatite-like layer on the outer enamel surface. This layer subsequently crystallizes into hydroxycarbonate apatite (HCA), closely resembling biological apatite both chemically and physically [65].

The enhancement of remineralization shown in SEM-EDX data from enamel pro varnish could be attributed to the abundance of released Ca and P ions from these products. The 5% NaF + ACP product elevated the levels of P, Ca, and F ions in saliva, preventing spontaneous precipitation and promoting the deep infiltration of these ions into subsurface lesions [55] (Fig. 4).

SEM-EDX results of Group 3 confirm the remineralization capability of the n-HAP that correlated with the PLM findings. It may be attributed to the n-HAP particles, which exhibit identical shapes, crystal structures, surface chemical compositions, and crystallinity to the apatite crystals in dental enamel. It was also demonstrated that crystal adhesion indicates stability and resistance to dissolution after PH cycling. Acidic conditions during PH cycling promote the solubility of n-HAP, thereby facilitating its deposition on the enamel surface., serving as a reservoir during pH fluctuations [66] (Fig. 5).

These results were consistent with the research by Daas et al., which found that n-HAP paste demonstrated a favorable protective effect on surface depositions and a smoother surface preservation than fluoride varnish over a longer duration after the pH cycle [44]. Kamath et al. (2017) also observed enhanced enamel texture in samples treated with n-HAP paste by SEM, results that agreed with our findings [67].

The present study found that the experimental 10% n-HAP varnish could remineralize artificial carious lesions, as determined by SEM-EDX analysis. This finding agreed with the research by Vijayasankari (2019), who also showed that the 10% experimental n-HAP paste had the best mineral gain and enhanced surface morphology results [49].

After pH cycling, the mean percentages of Ca, P, and F, as well as the Ca/P ratio, decreased in all groups. However, the decrease was significant in the Enamel Pro group and not substantial in the other three groups. The SEM-EDX results in the Enamel Pro group aligned with the PLM findings; it did not maintain adhesion to the enamel surface or extend remineralization during the pH cycle, likely due to the amorphous nature of the Enamel Pro group, in contrast to the n-BAG and n-HAP groups, which adhered to the enamel surface [65].

The EDX results revealed no significant changes between the n-HAP and n-BAG groups after the pH cycle. These results may be attributed to the reduced particle size and increased activity of n-HAP and n-BAG, which likely enhance the compound’s ability to penetrate the enamel surface and occlude the porosities of the artificial carious lesion more effectively than fluoride [9, 15].

**Fig. 9:**
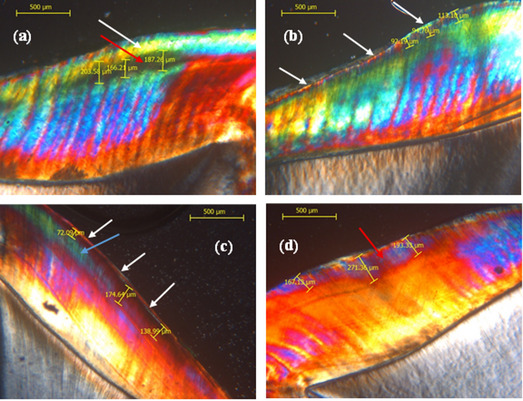
Polarized light micrographs of sectioned specimens. **A** Enamel treated with Enamel Pro varnish shows a minimal reduction in lesion body depth with evident positive birefringence (red arrow) and an increase in the highly mineralized surface layer (white arrow). **B** Enamel treated with nano-BAG varnish exhibits the lowest lesion depth, with an apparent remineralized surface layer (white arrow). **C** Enamel treated with nano-HAP varnish shows a surface remineralization band with negative birefringence (white arrow) and an evident reduction in lesion depth in certain areas (blue arrow). **D** Enamel treated with placebo varnish shows the greatest lesion depth, displaying positive birefringence and a distinct demineralized enamel band (red arrow)

The current study also showed that all commercial and experimental varnishes exhibited a notable decrease in lesion depth except for the control group. Additionally, the quality of the lesions among the groups differed, with the lowest lesion depth observed in the n-BAG group. These results aligned with the research by Hardikar, Anushka S., et al., who showed that the bioactive glass-incorporated varnish exhibited the most significant post-remineralization values across all groups for mineral content and lesion depth [11] (Fig. 9).

In contrast to our findings, Khandelwal et al. found that n-HAP toothpaste was slightly more effective than BAG. This result could be related to the different assessments, preparations, and formulations used, which may contribute to the inconsistency [68].

Primary enamel differs from permanent enamel in mineral composition, thickness, and surface structure. These differences can influence the response to remineralizing agents, as primary teeth may exhibit faster or more pronounced mineral changes due to their lower mineral density and thinner enamel [68, 69]. Therefore, the current results should be interpreted with consideration of the dentition type.

Our in vitro study had some limitations. Although the design ensured strict control of variables, it cannot accurately mimic the oral environment, even with artificial saliva. Factors such as saliva flow, enzymes, plaque biofilm, pellicle, chewing, brushing, and tongue movement affect varnish retention and effectiveness. Demineralization was chemically induced rather than by bacterial byproducts, which reduces clinical relevance. Only one concentration of the bioactive glass varnish was examined over a relatively short experimental period, which may not adequately reflect its long-term performance under oral conditions. Therefore, further studies with extended pH-cycling periods, larger sample sizes, and varying concentrations are needed to confirm the long-term efficacy of remineralizing materials in clinical settings, and to understand behavior, wear resistance, and varnish stability in simulated oral environments. The present study is limited by the absence of additional quantitative mechanical assessment methods, such as microhardness testing; therefore, future studies are recommended to incorporate such approaches to validate further and strengthen the findings. The current study used a colophony (rosin)-based varnish formulation. Future studies are also recommended to investigate the efficacy of these remineralizing agents when incorporated into gel- or toothpaste-based systems to further evaluate and compare their clinical performance. Another limitation was that lesion depth was not directly quantified after demineralization; instead, it was assessed visually using the ICDAS-II system, which correlates strongly with histological depth [70]. Subsequently, the results after remineralization and pH cycling were compared with those of a placebo varnish containing no active ingredients, as in previous investigations [41, 71].

## Conclusion

Within the limitations of the present study, it can be concluded that both experimental and commercial varnishes can improve the surface morphology and replenish the mineral content of artificial carious lesions. n-BAG varnish and n-HAP varnish demonstrated superior remineralization efficacy after 7-day pH cycling compared to Enamel Pro^®^ varnish, highlighting their potential as preventive agents for managing initial enamel caries in primary teeth.

## Supplementary Information

Supplementary Material 1.

## Data Availability

The datasets utilized and analyzed in this study can be provided by the corresponding author on reasonable request.

## Abbreviations

ACP: Amorphous calcium phosphate
CPP-ACP: Casein phosphopeptide-amorphous calcium phosphate
EDX: Energy Dispersive X-ray
HCA: Hydroxycarbonate apatite
n-BAG: Nano-bioactive glass
n-HAP: Nano-hydroxy apatite
PLM: Polarized light microscope
SEM: Scanning electron microscope
TEM: Transmission electron microscopy
XRD: X-ray diffraction
