# Supplementary material for: Assessment of the remineralizing potential of nano-bioactive glass, nano-hydroxyapatite, and sodium fluoride on artificial carious lesions in primary teeth
Source: BMC Oral Health. 2026 Apr 21;26:876. doi: 10.1186/s12903-026-08279-6 (PMC13192072; doi:10.1186/s12903-026-08279-6)
Supplement: Supplementary file 1 — Supplementary Material 1. [file 12903_2026_8279_MOESM1_ESM.docx]

**I-EDX analysis**

**1- Calcium weight percentage (Ca Wt.%)**

**1.1- (Intra-group comparison)**

Based on the Bonferroni Post Hoc results, the difference between the Baseline and Remineralization stages in the Enamel Pro group was not statistically significant. On the other hand, there were significant differences between the Demineralization stage and the other three stages, as well as between the pH stage and the other three stages. **In the Nano-BAG group,** significant differences were recorded between demineralization and the other three stages; however, no significant differences were detected between the baseline, remineralization, and after pH stages. In **the Nano-HAP group**, a significant difference was detected between the baseline and all subsequent stages, as well as between the Demineralization stage and the other three stages. However, no significant difference was recorded between the Remin and pH cycling stages. In contrast, **the Control group** exhibited significant differences between the baseline and all other stages; however, no significant differences were recorded between the Demineralization, Remineralization, and pH stages. The highly significant intra-group differences (P < 0.001) were confirmed for all four materials using ANOVA.

**1.2- (Inter-group comparison)**

The Nano-BAG group achieved the highest mean of Ca. Wt.% (42.65±4.14 %) after the remineralization stage, compared to the other three groups, while the Control varnish achieved the lowest mean (36.77±4.05 %) between the four groups at the same stage. Tukey's Post Hoc comparison revealed no significant variation in the mean Ca Wt.% between the four groups at baseline or after the demineralization stages. The Nano-BAG group revealed significant differences compared to the Control group after the Remineralization and pH cycling stages. Additionally, no significant differences were recorded between the Enamel Pro and Nano-HAP groups, nor between the Nano-HAP and Control groups.

The ANOVA test found a highly significant inter-group difference (P < 0.05) after the remineralization and pH cycling stages, while no significant differences were detected at baseline or following demineralization.

**Table 2** Mean ±SD, Intra, and Inter group comparison of Calcium weight Percentage (Ca Wt.%)

|  | **Baseline** | **After Demin** | **After Remin** | **After pH** | **P-value*** |
| --- | --- | --- | --- | --- | --- |
| **Enamel pro** | 41.7±4.56**^Aa^** | 34.38±1.35**^Ac^** | 41.21±2.84**^Aba^** | 38.1±2.29**^Abb^** | < 0.001**^HS^** |
| **Nano-BAG** | 43.05±2.63**^Aa^** | 35.34±2.16**^Ab^** | 42.65±4.14**^Aa^** | 41.03±1.91**^Aa^** | < 0.001**^HS^** |
| **Nano-HAP** | 42.07±4.37**^Aa^** | 35.14±2.12**^Ac^** | 38.77±3.05**^BCb^** | 38.88±1.77**^Abb^** | < 0.001**^HS^** |
| **Control** | 40.86±2.6**^Aa^** | 36.41±1.91**^Ab^** | 36.77±4.05**^Cb^** | 35.43±1.91**^Bb^** | < 0.001**^HS^** |
| **P-value**** | **0.566^NS^** | **0.113^NS^** | **< 0.001^HS^** | **< 0.001^HS^** |  |

-* Overall P-value for Intra-group comparison between the four time intervals (Repeated Measures ANOVA test).

-** Overall P-value for Inter-group comparison between the four groups (ANOVA test).

- Small letters for pairwise comparison between different time intervals, while the capital letters are for pairwise comparison between groups. There is no significant difference between means that share at least one superscript letter at a significance level of P ≤ 0.05.

- S= Statistically significant at P ≤ 0.05 - NS= Non-significant P < 0.05.

- HS = Highly significant at P ≤ 0.001

**2- Phosphorus (P Wt.%)**

**2.1- (Intra-group comparison)**

Concerning the Bonferroni Post Hoc results**,** no significant differences were detected between the four stages for **the Enamel pro group**. Similarly, the difference between the four stages was insignificant for **the Nano-BAG group**. **For the Nano-HAP group,** a significant difference was found between the Demineralization and Remineralization stages; however, no significant difference was detected between the other three stages. **For the Control group,** significant differences were detected between the first two stages (Baseline and Demineralization) and the subsequent two stages (Remineralization and pH cycling stages). In contrast, no significant differences were recorded between the Baseline and Demineralization stages and between the Remineralization and After pH stages.

The ANOVA test revealed a significant intra-group difference (P < 0.05) in the Control and Nano-HAP groups, but not in the Enamel Pro and Nano-BAG groups.

**2.2- (Inter-group comparison)**

We can conclude the following: Although the Nano-BAG group achieved the highest mean of P Wt.% (18.5±1.31 %) after the Remineralization stage, no statistically significant differences were detected among the four groups at any of the four stages (Baseline, Demineralization, Remineralization, or pH cycling). The overall P-value for inter-group comparisons was not significant (P > 0.05) at any of the four stages.

**Table 3** Mean ±SD, Intra, and Inter group comparison of Phosphorus weight Percentage (P Wt.%)

|  | **Baseline** | **After Demin** | **After Remin** | **After pH** | **P-value*** |
| --- | --- | --- | --- | --- | --- |
| **Enamel pro** | 18.49±0.71**^Aa^** | 19.74±0.82**^Aa^** | 18.1±0.66**^Aa^** | 19.13±1.66**^Aa^** | **0.053^NS^** |
| **Nano-BAG** | 19.25±0.73**^Aa^** | 19.35±1.32**^Aa^** | 18.5±1.31**^Aa^** | 19.13±0.91**^Aa^** | **0.112^NS^** |
| **Nano-HAP** | 18.46±1.21**^Aab^** | 19.75±1**^Aa^** | 17.36±1**^Ab^** | 19.63±1.19**^Aa^** | **0.013^S^** |
| **Control** | 19±1.35**^Aa^** | 19.48±0.84**^Aa^** | 17.51±1.01**^Ab^** | 17.98±1.83**^Ab^** | **0.009^S^** |
| **P-value**** | **0.265^NS^** | **0.759^NS^** | **0.479^NS^** | **0.621^NS^** |  |

There is no significant difference between means that share at least one superscript letter at a significance level of P ≤ 0.05.

- S= Statistically significant at P ≤ 0.05 - NS= Non-significant P < 0.05.

- HS = Highly significant at P ≤ 0.001

**3- Fluoride (F Wt.%)**

**3.1- (Intra-group comparison)**

According to the Wilcoxon signed-rank test, **for the Enamel pro group,** the mean of F Wt.% % significantly increased after the Remineralization stage, and a significant difference was revealed between the Remineralization stage and the Demineralization & pH stages. On the other hand, there were also no significant differences between the baseline and the other three stages, nor between the Demineralization and pH stages. **For the Nano-BAG group,** significant differences were detected between the baseline and the other three stages; however, no significant difference was found between the Demineralization, Remineralization, and After pH stages. **For the Nano-HAP group,** significant differences were observed between the baseline and demineralization stages; in contrast, no significant differences were detected between the baseline, remineralization, and pH stages. Also, no significant difference was revealed between the Demineralization, Remineralization, and pH stages. A substantial difference was revealed between the baseline and the subsequent three stages **in the control group**, although no significant differences were identified in the demineralization, remineralization, and pH stages. Significant intragroup differences were detected among the four groups (P < 0.05) using the Friedman test**.**

**3.2- (Inter-group comparison)**

No significant changes in the mean of F Wt.% % were detected between the four groups at any stage, as indicated by the Mann-Whitney test. Based on the Kruskal-Wallis test, the inter-group comparison's overall P-value was not statistically significant at any stage (P > 0.05).

**Table 4** Mean ±SD, Intra, and Inter group comparison of Fluoride weight Percentage (F Wt.%)

|  | **Baseline** | **After Demin** | **After Remin** | **After pH** | **P-value*** |
| --- | --- | --- | --- | --- | --- |
| Enamel pro | 0.39±0.4**^Aab^** | 0±0**^Ab^** | 0.81±0.97**^Aa^** | 0.16±0.19**^Ab^** | **0.025^S^** |
| Nano-BAG | 0.39±0.54**^Aa^** | 0±0**^Ab^** | 0.1±0.32**^Ab^** | 0.1±0.22**^Ab^** | **0.036^S^** |
| Nano-HAP | 0.68±0.73**^Aa^** | 0±0**^Ab^** | 0.27±0.41**^Aab^** | 0.21±0.42**^Aab^** | **0.011^S^** |
| Control | 0.6±0.68**^Aa^** | 0±0**^Ab^** | 0.2±0.39**^Ab^** | 0.02±0.06**^Ab^** | **0.008^S^** |
| P-value** | **0.648^NS^** | **1.000^NS^** | **0.077^NS^** | **0.082^NS^** |  |

There is no significant difference between means that share at least one superscript letter at a significance level of P ≤ 0.05.

- S= Statistically significant at P ≤ 0.05 - NS= Non-significant P < 0.05.

- HS = Highly significant at P ≤ 0.001
